# Glucose variability in 6–12-month-old healthy infants

**DOI:** 10.3389/fnut.2023.1128389

**Published:** 2023-07-12

**Authors:** Michael Hauschild, Cathriona Monnard, Alison L. Eldridge, Maria Christina Antoniou, Thérèse Bouthors, Erik Hansen, Andrew A. Dwyer, Andreas Rytz, Christian Darimont

**Affiliations:** ^1^Pediatric Endocrinology, Diabetes and Obesity Unit, Department Woman-Mother-Child, Lausanne University Hospital and University of Lausanne, Lausanne, Switzerland; ^2^Nestlé Institute of Health Sciences, Nestlé Research, Lausanne, Switzerland; ^3^Boston College, William F. Connell School of Nursing, Chestnut Hill, MA, United States; ^4^Clinical Research Unit, Nestlé Research, Lausanne, Switzerland

**Keywords:** glucose variability, infants, nutrition, circadian rhythms, Continuous Glucose Monitoring (CGM)

## Abstract

**Background:**

Metabolic programming of glucose homeostasis in the first 1,000 days of life may impact lifelong metabolic and cardiovascular health. Continuous glucose monitoring (CGM) devices may help measure the impact of dietary intake on glucose rhythms and metabolism in infants during the complementary feeding period.

**Objectives:**

Demonstrate the feasibility of CGM to measure and quantify glucose variability in response to infant feeding and to evaluate associations between macronutrient meal composition and glucose variability.

**Methods:**

The “FreeStyle Libre Pro^®^” device interstitial glucose meter was applied to the anterior thigh of 10 healthy 6–12-month-old infants. Parents recorded food intake, time of feeding, and used daily dairies to record sleep time and duration. Descriptive statistics were employed for food intake, sleep and key glycemic parameters over three full days. Mixed linear models were used to assess glycemic changes.

**Results:**

Mid-day, afternoon, and evening feeds contained >30 g carbohydrate and induced higher 2-h iAUC (3.42, 3.41, and 3.50 mmol/L*h respectively) compared to early and mid-morning feedings with ≤25 g carbohydrates (iAUC 2.72 and 2.81 mmol/L*h, *p* < 0.05). Early morning and evening milk feedings contained approximately 9 g of fat and induced a longer time to reach maximal glucose value (Tmax; 75 and 68 min, respectively) compared to lower fat feedings (2.9–5.9 g; Tmax range: 34–60 min; *p* < 0.05). Incremental glucose value at time of food intake (C0) increased significantly from 0.24 ± 0.39 mM in early morning to 1.07 ± 0.57 mM in the evening (*p* < 0.05). Over the day, 70% of glucose values remained within the normal range (3.5–5.5 mmol/L), 10% were between 5.5–10 mmol/L, and 20% were < 3.5 mmol/L.

**Conclusion:**

Our data support the feasibility of using CGM to measure glucose in 6–12-month-old infants. The observation of possible diurnal glucose variability and typical glucose values may have implications for future studies investigating metabolic adaptation to nutritional intake in early life.

## 1. Introduction

Early nutrition and the introduction of complementary feeding represents a key physiological step in early life when infants transition from a fully milk diet to solid foods. During this time, the foundations for healthy growth and metabolic health later in life are established (1). One indicator of metabolic health might be the physiologic variability of blood glucose in response to feeding. Glucose variability (GV) has been linked to endothelial dysfunction (2) and clinical studies support the role of GV in the evolution of vascular complications of diabetes (3).

The potential link between glucose variability before the age of 2 years and subsequent pediatric or adult disease onset emphasizes the need to examine glucose response to normal nutrition in early infancy. Studies of glucose rhythms in preterm infants (4–8) provided some insights into glucose regulation in early life. In healthy, term infants, limited studies have measured blood glucose and insulin concentrations in response to different infant feeding practices and the impact on metabolism in early life (9–11). More recently, Kouwenhoven et al. (12) observed that formula-fed infants were less insulin sensitive compared to breastfed infants. However, beyond the early milk-feeding period (0–6 months) there is a significant gap in the literature on glycemic responses in healthy, term infants and young children up to 2 years of age.

Continuous glucose monitoring (CGM) systems offer a novel, less invasive approach to continuously measure glucose concentration in the interstitial fluid of subcutaneous tissues. Notably, CGM measures directly correlate with capillary blood glucose levels in adults (13). Such CGM systems have been used to evaluate accuracy and clinical use in specific groups like children with diabetes (14, 15), preterm infants (4, 5, 16) as well as neonates with transient hyperinsulinism (17). However, little is known about normal daily fluctuations or rhythms in plasma glucose concentrations in healthy infants during the complementary feeding period.

The primary aim of this proof-of-concept study was to demonstrate that glucose excursions in response to infant feeding can be measured and quantified using CGM interstitial glucose measurements in healthy 6–12-month-old infants. We also aimed to characterize daily blood glucose fluctuation patterns in response to feeding periods and sleep. Finally, we sought to examine the impact of the type and composition of foods and beverages on glucose variability in infants. Our study did not aim to validate CGM in this age group. A better understanding of GV in infants could be used for disease prevention, diagnosis or monitoring of the growth rate and metabolic health in later years, and to inform nutritional strategies to support healthy growth and development as well as for preventing cardiovascular diseases in adulthood.

## 2. Methods

This was an observational, non-intervention exploratory study in healthy full-term infants 6–12 months old. Healthy infants whose parents agreed to participate in the study were recruited from clinic visits at the Lausanne University Hospital (Centre Hospitalier Universitaire Vaudois, CHUV, Lausanne, Switzerland). Participants met the following inclusion criteria: (i) full-term (between 37 and 42 weeks) single infant, (ii) 6 to12-months-of-age, (iii) in good general health, (iv) fed with a variety of foods including partial breastfeeding, commercial infant formulas, plus solid foods such as cereals, grains, meats, fruits, and vegetables. Exclusion criteria were (i) premature birth (<37 weeks gestation), (ii) child whose parent had a diagnosis of type 2 diabetes or mother with gestational diabetes, (iii) fully breastfed child (i.e., no commercial infant formula), (iv) chronic skin conditions such as eczema or excessive dryness, (v) known allergy to adhesive bandages/plasters, (vi) evidence of infectious diseases affecting the skin (e.g., chicken pox, scarlet fever), and (vii) disorders affecting skin microcirculation.

For CGM, we used the FreeStyle Libre Pro^®^ (Abbott^®^ Diabetes Care, Witney, Oxon, UK). This system meets the accuracy criteria specified in the final draft ISO 15197 and POCT12, with 97.3 to 98.9% of the individual results of various blood sample types. The device utilizes a previously described glucose oxidase measurement assay (15). The FreeStyle Libre Pro^®^ interstitial glucose meter was applied by a physician at the initial study visit after preparing the skin with a topical lidocaine and prilocaine- anesthetic cream (Emla^®^). Due to physical constraints, the device was applied on the infant’s anterior thigh. Measurements were recorded over a total of 5 days while the infants were at home. Parents received a card with details of the device and contact numbers in case of questions or problems. Data were retrieved at the end of the sensor-wearing period using a dedicated reader. Parents were unable to access the sensor data while the sensor was in place on the infant. No capillary or venous blood glucose measurements were taken during this study to minimize the invasive procedures in this healthy cohort. Data collected during the first and last day were not included in our analysis.

### 2.1. Nutritional and sleep assessment

Infants followed their usual diet without modification. To accurately assess relationships between macronutrient consumption and interstitial glucose fluctuations, parents kept daily diaries and recorded the time of each feeding, name of the formula and/or complementary foods, amount consumed, as well as the duration of the feeding period or the ingestion of complementary medication (children’s and mothers). The investigators reviewed food and sleep diary with the parents to ensure data consistency. No data were removed from analysis due to non-completion.

For breastfed children, we recorded the time of feeding but not the amount. For branded products, macronutrient content information was obtained from product labels. For other foods, we obtained information on nutrient content from the Swiss Food Composition Database.^1^ In situations when no entries were found in the Swiss database, we retrieved nutrient composition from the United States Department of Agriculture Food Data Central.^2^ Parents also used the diary to record infant day/night sleeping times and duration by direct observation.

### 2.2. Statistical analysis

No data are available on glucose variability in healthy infants during the complementary feeding period. Thus, no formal power calculations could be performed. In healthy adults, the standard method for determining the glycemic index of carbohydrates in food (ISO26642)^3^ uses data on 10 individuals for robust estimates of key glycemic parameters including: incremental area under the curve (iAUC), incremental maximal glucose value (Cmax) and time to reach this value (Tmax) – per the International Organization for Standardization 2010, ISO 26642:2010 food products determination of glycemic index (GI) and recommendation for food classification. Consequently, we set our sample size to 10 infants for this proof-of-concept observational study.

Data analysis was primarily descriptive (i.e., range, median, mean, standard deviation). Participant characteristics, including age and anthropometrics at study start, and birth measures were tabulated using mean ± standard deviation (SD) median and range for the 10 infants. Similarly, parameters of the sleep (i.e., number of sleeping occasions over 24 h, sleeping duration, start time of sleeping occasions) are reported using mean ± SD (Table 1).

**Table 1:** Age, birth characteristics, anthropometrics, sleep and eating occasions (mean over three consecutive days) of the *N* = 10 infant participants including 5 females and 5 males (Range, Median, Mean ± SD) (Nb = number).

Characteristic	Range Min – Max	Median	Mean ± SD
Age (months)	7.6–12.0	9.9	10.2 ± 1.5
Gestation (weeks)	38.3–42.0	40.5	40.3 ± 1.2
Birth weight (kg)	2.73–4.77	3.45	3.63 ± 0.72
Birth length (cm)	47.0–55.0	50.8	50.8 ± 2.5
Length (z-score)	−1.24 to 3.26	1.09	0.97 ± 1.27
Weight (z-score)	−2.37 to 1.27	0.42	0.26 ± 1.08
BMI (z-score)	−2.34 to 0.87	−0.22	−0.39 ± 1.17
Overall sleep occasions (Nb/24 h)	2.3–3.0	2.7	2.7 ± 0.3
Overall sleep duration (h/24 h)	11.8–14.1	13.1	13.0 ± 0.7
Night sleep duration (h/night)	9.7–12.3	10.7	10.8 ± 0.7
Morning sleep duration (h/morning)	0.2–1.9	1.0	1.1 ± 0.7
Afternoon sleep duration (h/afternoon)	0.5–1.7	1.2	1.1 ± 0.4
Sleep start night (hh:mm)	19:10–21:55	20:25	20:23 ± 46
Sleep start morning (hh:mm)	08:10–11:45	09:56	09:47 ± 59
Sleep start afternoon (hh:mm)	13:00–16.53	14:18	14:27 ± 55
Eating occasions (Nb/24 h)	3.7–7.0	5.0	5.2 ± 1.0

Food diary data were collected from the day the CGM device was placed (day 1) to the day the device was removed (day 5). Food intake was recorded from the first to the last day of participation (day 1 to 5). Three full, consecutive days of intake were collected for each infant (days 2 to 4), corresponding to the 72 h period of continuous glucose monitoring. We disregarded the first day glucose values due to the lower accuracy during this initial period. Basic food intake parameters (i.e., number of eating occasions over 24 h, starting time of feeding) are reported using mean ± SD. The distribution of prevalence of eating and drinking occasions over the 24 h were used to create 6 discrete periods (Supplementary Figure S1). These periods are early morning (06:00–08:00), mid-morning (08:00–11:00), mid-day (11:00–14:30), afternoon (14:30–18:00), evening (18:00–24:00), and night (00:00–06:00). Energy and macronutrient intakes from feeding occasions are reported using mean ± SD. Mean glucose load was estimated for each eating occasion (18).

**Figure 1:**
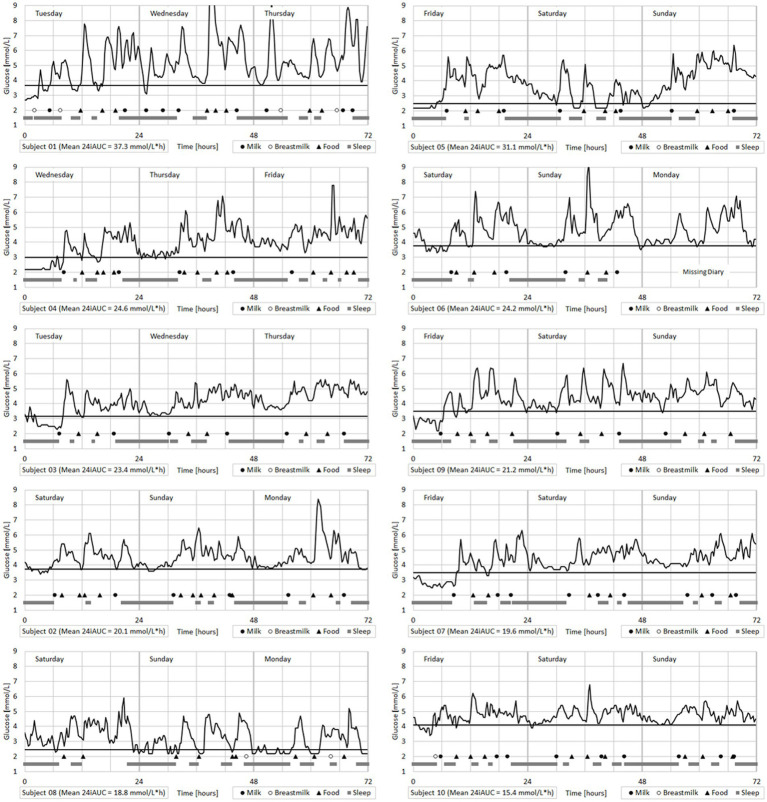
Individual infant glucose monitoring over 72 h with sleep times and food intakes reported from diaries.

**Figure 2:**
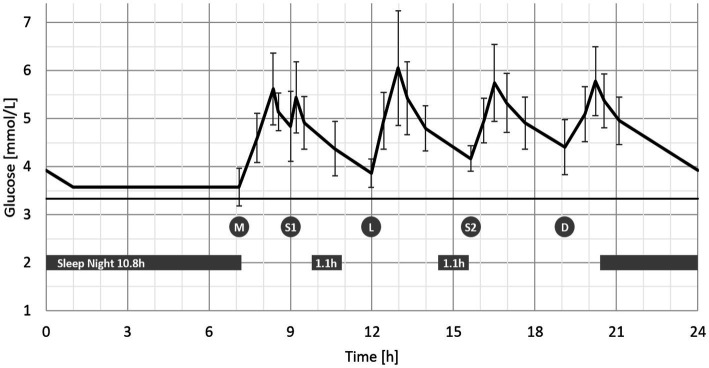
Average glucose curve in infants over 24 h^1^ with sleep and food intake patterns (Mean ± SD, *N* = 10). ^1^Feeding occasions are abbreviated as follows: M – early morning, S1 – mid-morning, L – mid-day, S2 – afternoon, D – evening.

Feeding events were tabulated in parallel with CGM parameters. The distribution of glucose values and glucose variability (coefficient of variation, CV) is reported for both 24 h as well as main feeding events. Individual baseline glucose levels were determined to derive common parameters of postprandial glucose response. As overnight fasting cannot be imposed to infants, the baseline glucose value was computed as the 3-day mean of the 25th percentile of recorded nighttime values (00:00–06:00). The 25th percentile was preferred over a measure of central tendency (such as arithmetic mean or median) to preclude bias introduced by the rare occasions when infants received night feedings. After calculating the baseline glucose, we derived incremental glucose value at time of food intake (incremental iC0), maximal increase after food intake (incremental Cmax), and time to reach this value (Tmax). The time to return to baseline (TbackBL) could only be calculated after the evening feeding – as glucose values almost never returned to baseline during the day, possibly due to the relatively high meal frequency. The incremental area under the curve (both 2 h postprandial and total over 24 h) was estimated using the trapezoid method of each individual curve. All dependent variables Y listed in Table 2 were analyzed using a mixed linear model for repeated measures with independent variables being (A) eating occasions as fixed, (B) infants as random and (C) days as nested within infants, leading to the model Y ~ A + B + C(B) + Error. In cases where the factor eating occasion was significant (*p* < 0.05), pairwise comparisons of eating occasions were performed using Fisher’s Least Significant procedure, using a significance level of α = 5% (LSD5%). Consequently, two feeding events differing by more than LSD5% can be considered significantly different (*p* < 0.05), without considering any multiplicity correction (due to the exploratory nature of the proof-of-concept study). Consequently, the standard deviation (SD) represents between-subject variability for an average 24 h monitoring period. Individual data including sleep occasions, feeding events and glucose monitoring are visualized over 72 h with the 10 subjects being ordered by decreasing 24 h-iAUC (Figure 1). Average timing and duration of sleep occasions and feeding events are visualized together with a prototypical average glucose curve over 24 h (Figure 2). To be representative, this prototypical average curve features five points per eating occasion. These are the two tabulated glucose levels corresponding to the time T0 and Tmax (Table 2), as well as three additional timepoints corresponding to T2h, the time to reach the average of C0 and Cmax and the time to reach the average of Cmax and C2h.

**Table 2:** Time of intake, nutrient intake, glucose ranges, and glucose parameters over 24 h and per eating occasion (mean over three consecutive days) of the *N* = 10 infant participants (Mean ± SD).

	24 h	Early morning	Mid-morning	Mid-day	Afternoon	Evening
		6:00–8:00	8:00–11:00	11:00–14:30	14:30–18:00	18:00–24:00
Food diary data						
Feeding start time (hh:mm)		07:07 ± 59	08:39 ± 31	11:58 ± 46	15:38 ± 41	19:06 ± 29
Energy (kcal)	822 ± 264	191 ± 66	81 ± 68	262 ± 128	191 ± 95	264 ± 82
Carbohydrates (g)	112.5 ± 43.8	23.6 ± 8.8	10.6 ± 7.0	37.9 ± 19.8	30.7 ± 13.5	36.8 ± 13.9
Total sugars (g)	59.6 ± 21.9	20.1 ± 7.8	5.0 ± 5.2	13.2 ± 7.1	22.8 ± 9.9	19.1 ± 7.9
Protein (g)	25.4 ± 12.7	3.7 ± 1.3	2.6 ± 2.7	11.9 ± 7.8	4.5 ± 2.4	7.6 ± 3.9
Fat (g)	25.4 ± 7.4	8.9 ± 3.0	2.9 ± 3.4	5.9 ± 2.8	5.0 ± 4.0	9.0 ± 2.3
Fibers (g)	9.5 ± 5.8	1.2 ± 1.2	1.1 ± 1.1	5.2 ± 2.2	2.5 ± 1.6	2.8 ± 2.9
Estimated glucose load (g)	62.1 ± 26.0	10.7 ± 4.5	6.0 ± 3.2	22.3 ± 12.6	15.5 ± 7.1	20.0 ± 10.3
Glucose measurements by CGM						
Baseline (mmol/L)	3.34 ± 0.55					
Incremental 24-AUC (mmol/L * h)		2.72 ± 1.04	2.81 ± 0.89	3.42 ± 1.36	3.41 ± 1.05	3.50 ± 1.25
Incremental Cmax (mmol/L)		2.28 ± 0.74	2.11 ± 0.74	2.72 ± 1.20	2.41 ± 0.80	2.44 ± 0.72
Tmax (min)		75 ± 27	34 ± 24	60 ± 36	52 ± 21	68 ± 21
Incremental C0 (mmol/L)		0.24 ± 0.39	0.90 ± 0.82	0.53 ± 0.29	0.83 ± 0.27	1.07 ± 0.57

### 2.3. Ethical considerations

The clinical trial was approved by the Cantonal Commission on Ethics in Human Research (CER-VD) CER-VD/2017-02090, as well as the Swiss Agency for Medical Devices Swissmedic reference number: 10000129 and EUDAMED reference number: CIV-18-07-024927. The study was registered with ClinicalTrials.gov Identifier: NCT03819725. The project leader confirms that the project was conducted according to the approved protocol, the Swiss legal requirements, the current version of the World Medical Association Declaration of Helsinki and the principles of Good Clinical Practice.

## 3. Results

### 3.1. Subjects

Out of 54 infants screened, 10 infants were recruited (50% females). Personal or organizational reasons were given by 60% of those who declined to participate; other reasons included unwillingness to participate in studies or fear. The flow diagram is provided as part of the Online Supplementary material. At enrollment, infants were 10.2 ± 1.5 months old with normal weight (*z*-score: 0.26 ± 1.08) and length (*z*-score: 0.97 ± 0.27) (Table 1). All infants completed the 5-day observation time. Two subjects developed local skin reactions (classified as adverse events) noted on device removal, yet both resolved rapidly without intervention. No serious adverse events were observed. Parents did not report any problems with the CGM device while bathing their infant. Similarly, parents did not report any signs of infant discomfort, sleep disturbance, or unusual behavior attributable to CGM device placement. The number of sleep occasions and duration were quite consistent across infants. Infants generally slept 10.8 ± 0.7 h at night with naps (approximately one hour) in the morning and afternoon.

### 3.2. Feeding

Infants received a median of five (3.7–7.0) feeding events per day, and feeding patterns were quite similar across infants (Table 2). The first eating occasion consisted in >90% of infant formula or breast milk. On 40% of presorted days, some type of light feeding occurred mid-morning, consisting of fruit, bread, baby biscuits (cookies) or infant cereal. The mid-day feeding included both home-prepared and commercial baby foods that generally included a variety of vegetables, protein-rich foods (e.g., meats, fish, chicken) plus potatoes, pasta or rice. Most days (83.3%), infants were fed during the afternoon, usually with fruits and yoghurt. In the evening, most infants were fed infant formula and/or breast milk (Supplementary Figure S3). Two infants received breast milk during the night. In total, breastfeeding events represented 7 out of 64 (11%) milk intakes. One infant was breastfed on one night, and the other was breastfed on two nights. Breastfeeding mothers did not report ingestion of medications. There were six other occasions (among three infants) when infant formula feeding occurred between 05:15–05:50, slightly earlier than the ‘morning’ feeding period. The early morning and evening feedings mostly comprised infant formula or breast milk and contributed the greatest amount of fat. In contrast, the mixed ‘mid-day’ feeding contributed the most protein and dietary fiber. The ‘mid-day’ and ‘evening’ feeding periods were similar in daily energy contribution (Table 2).

### 3.3. Glucose fluctuation analysis

In all infants, each feeding event was associated with a significant increase in interstitial glucose (Figure 1; Table 2). Feeding events with carbohydrate contents >30 g (i.e., ‘mid-day,’ ‘afternoon,’ and ‘evening’) induced higher 2 h-iAUC (3.42 ± 1.36, 3.41 ± 1.05, and 3.50 ± 1.25 mmol/L*h respectively) compared to the two morning-feedings that contained <25 g of carbohydrates (iAUC: 2.72 ± 1.04 and 2.81 ± 0.89 mmol/L*h respectively). The difference between higher (>30 g) and lower (<25 g) carbohydrate feedings was statistically significant (*p* < 0.05, LSD5% = 0.60 mmol/L*h). The same trend was observed for incremental Cmax, but only ‘mid-day’ (2.72 ± 1.20 mmol/L) vs. the two ‘morning’ occasions (2.28 ± 0.74 and 2.11 ± 0.74 respectively) were statistically significant (LSD5% = 0.42 mmol/L).

Feeding events that included milk (‘early morning’ and ‘evening’) contained higher fat content (approximately 9 g) and induced a larger Tmax (75 ± 27 and 68 ± 21 min respectively) compared to the three other occasions containing between 2.9 and 5.9 g fat (Tmax: 34 ± 24, 60 ± 36, and 52 ± 21 min respectively, *p* < 0.05, LSD5% = 16 min).

Incremental C0, representing the difference between glucose values before each excursion and the fasting glucose value (3.34 ± 0.55 mM), increased progressively during the day from 0.24 ± 0.39 mM in ‘early morning’ to 1.07 ± 0.57 mM in the ‘evening’ (*p* < 0.05, LSD5% = 0.31 mmol/L) (Table 2). In addition, glucose excursion induced by the ‘evening’ feeding, starting on average at 19:06 ± 29 min, returned to baseline at 00:18 ± 53, more than 5 h later.

### 3.4. Glucose ranges

Glucose values from observation days 2 to 4 were analyzed, revealing 70% of values within the normal range (3.5–5.5 mmol/L) (19), 10% measured in the 5.5–10 mmol/L range, 0% in the >10 mmol/L range, and 20% in the <3.5 mmol/L range (with 11% in the <3.0 mmol/L range). Examining the overnight steady-state glucose values, the distribution of CGM-measured glucose values was strikingly different, with up to 34% of glucose values measuring <3.5 mmol/L, and 15% values measured <2.6 mmol/L.

## 4. Discussion

The data presented herein demonstrate the feasibility of using CGM to detect and measure glucose fluctuations throughout the day in infants 6–12 months of age. The CGM device was well tolerated by all study participants. Analysis of individual subject data clearly showed the impact of feeding occasions on glucose excursions.

Due to the proof-of-concept design of our study, neither standardized meals nor strict between-meal intervals were imposed. However, overall food intake was similar to those reported for 6–12-month-olds in the U.S. Feeding Infants and Toddlers Study (FITS) (20). In the present study, parents completed diet diaries. In contrast, the FITS study relied on interviews to collect 24-h food recalls. Nevertheless, energy intake observed in this study (822 ± 264 kcal/d) was similar to levels reported in FITS (852 kcal/d). This slight difference may be attributable to inaccurate reporting (i.e., missed items) in the diaries and/or inaccuracies in food recall. Daily carbohydrate intake in our study (112.5 ± 43.8 g/d) was also similar to intakes reported in FITS (111 g/d).

Metabolic responses in infancy can be modulated by exogenous factors, including diet (10). In the current study, we calculated the macronutrient composition of each meal. We observed that intake of carbohydrate and sugars corresponded with greater increases in 2 h iAUC and incremental Cmax values, while higher fat content in the ‘morning’ and ‘evening’ meals corresponded to greater Tmax responses. There is limited evidence regarding the impact of macronutrients on glycemic response in infants. Some studies have explored glycemic response to milk in the first 6-months-of-life (11, 12, 21), showing differences between human breast milk and infant formula, which may reflect the documented compositional differences between the two. Fleddermann et al. investigated the impact of carbohydrate quality on glycemic response over 4 weeks in 4–8-month-old infants and found higher plasma glycemia in response to a formula containing isomaltulose compared to a conventional isocaloric formula containing maltodextrin (9). In another study, significantly higher glucose levels were observed in 3-month-old infants consuming a lactose-based formula compared to a lactose-free formula containing corn-syrup solids (10). With respect to our observation of increased Tmax values in the ‘morning’ and ‘evening,’ it is plausible that the increased fat content consumed at these two timepoints (compared to other eating occasions with different macronutrient composition) modulated the glycemic response, resulting in greater Tmax values. Indeed, previous studies in infants have demonstrated the effect of fat on delayed gastric emptying (22–25), and we cannot rule out an effect of the relatively high meal frequency. In adults, delayed gastric emptying has been shown to lower the glycemic response to carbohydrate-based foods (26). In our study, due to the limited number of breastfeeding occasions (11%), no comparison of breastfeeding and formula feeding was performed. Moreover, breastmilk composition was not measured in this study.

As discussed above, we did not administer standardized meals due to our study approach. The lack of fixed intervals between eating occasions and the lack of fixed macronutrient composition of meals may have impacted iC0 and iCmax results. However, we performed a detailed analysis of the first and last feeding events of the day, which consisted primarily of milk. We observed a slightly higher glucose AUC in the mid-morning period as compared to the early morning period and describe a prolonged (5 h) return to the basal glucose level following the ‘late evening’ feeding. Data of mixed meal tolerance and OGTT data in children suggest a return to fasting glucose values after 120–180 min (27) and a peak glucose and insulin peak at 30 min (28). In insulin-resistant obese children, prolonged times (up to 240 min) have been observed (29, 30), but data for very young children are missing. Our findings might suggest the possibility of differences in metabolism and insulin sensitivity throughout the day in 6-12-months-old infants, similar to observations in adults (31, 32). However, further studies, including standardized meals, measurements of metabolic parameters like insulin, and in-depth comparison between breastfeeding and infant formula, are needed to support such claims.

Overall, our study findings suggest that differences in infant diet composition impact early life metabolism. Further, our observations suggest that feeding patterns may play a role in the metabolic response to macronutrient intake in early life. Baseline glucose values (iC0) were higher at the end of the day, and we observed a clear stabilization of the values during the night. To our knowledge, this is a unique observation that has not been described previously and points to possible circadian rhythms in glucose utilization in infants 6–12 months old, but other aspects like the time to return to the true baseline must be considered.

The observation of differences in baseline rhythms during daytime and nighttime and the progressive increase of C0 is consistent with the existence of circadian rhythms as early as in the first year of life. Diurnal variations in insulin sensitivity and glucose metabolism have been described since 1972 (33). In a recent review, Stenvers et al. detailed possible links between the central clock and glucose metabolism, sensitivity and insulin secretion (34). Several studies suggest possible misalignments between the central clock and daily rhythms of sleep with disturbed glucose metabolism (35). As the population in our proof-of-concept study consisted of normal infants with normal daily sleep and feeding periods, other aspects like the time to return to the true baseline must be considered.

We found a relatively high percentage of time when infants had glucose values <3.5 mmol/L – levels that are typically considered hypoglycemic in this age group. Moreover, we observed a high percentage of time when glucose values <3.0 mmol/L and even <2.6 mmol/L during the night. Our study did not aim to validate CGM in this age group and therefore are cautious about the interpretation of these results. Furthermore, we are aware of the difficulty to interpret the values due to the lower accuracy and precision of CGM devices in low glycemic ranges as published for preterm infants (36), but the observed levels probably reflect physiological low glucose values in this age group. Our observations suggest the need to revise the normal blood glucose ranges for healthy infants <1 year of age. Apart from the neonatal period, fasting blood glucose levels of 3.5–5.5 mmol/L are usually considered normal for infants, children, and adults (19). However, there is a paucity of data on glucose response in children under 1 year-of-age. Low blood glucose levels (2.6–3.4 mmoL/L) are considered normal in newborns and breastfed newborns tend to have significantly lower values than formula-fed newborns (37). Our study findings further indicate that lower normal ranges during nighttime should be considered during therapeutic interventions (or follow-up) of very young children with specific metabolic diseases like type 1 diabetes.

This minimally invasive proof-of-concept study was not intended to evaluate the accuracy of the CGM device, and therefore no paired blood glucose values were measured. Specifically, we did not validate individual CGM values for 6–12 month-olds by conducting parallel measurements in blood samples. We conducted the study under the assumption that glucose values reported by a factory-calibrated device are within the acceptable reliability ranges as previously demonstrated in young infants aged 30–140 days (38), 2–8 year-old children (39) and older children (15). Moreover, the accuracy of CGM, as measured by the mean absolute relative difference (MARD), is above the recommended value of 10% during hypoglycemia in preterm infants (36). Limitations of the exploratory observational study include the small sample size and relative lack of dietary diversity. Moreover, the mix of breast- and formula-fed infants included in this study does not allow for a true exploration of circadian rhythms. Future studies are required with a larger sample size, separating breast- and formula-fed infants to compare feeding methods and to determine the impact of maternal milk composition and timing on circadian patterns of metabolism.

In conclusion, our study shows that CGM devices like the Freestyle Libre^®^ Pro are a safe and useful tool to study glucose metabolism in young children. Using CGM measurements, we showed the impact of timing of food consumption and macronutrient intake on postprandial glucose response in infants 6–12 months old and confirmed that glucose levels respond differentially to different macronutrient compositions. To the best of our knowledge, our observations suggest for the first time the possibility of diurnal glucose variability and the presence of circadian rhythms in infants 6–12 months of age. One relevant implication of our work might be the use of CGM devices for an etiological understanding of pediatric or adult-onset diseases, like studying glucose metabolism in infants born from mothers with type 2 diabetes or obesity. In addition, our data raise questions regarding the widely accepted normal glucose ranges for this age group. Further studies using CGM devices and standardized meal protocols in infants are needed to confirm our observations and explore the impact of complementary feeding on glucose metabolism.

## Data availability statement

The original contributions presented in the study are included in the article/Supplementary material, further inquiries can be directed to the corresponding author.

## Ethics statement

The clinical trial was approved by the Cantonal Commission on Ethics in Human Research (CER-VD) CER-VD/2017-02090, as well as the Swiss Agency for Medical Devices Swissmedic reference number: 10000129 and EUDAMED reference number: CIV-18-07-024927. The study was registered with ClinicalTrials.gov Identifier: NCT03819725. Written informed consent to participate in this study was provided by the participants’ legal guardian/next of kin.

## Author contributions

MH, CM, AR, and CD designed the research. MH, MA, TB, and EH recruited participants and conducted clinical visits. AR prepared datasets and performed the statistical analysis. AE assessed dietary intakes from participant diaries. MH, CM, AE, AR, AD, and CD wrote the manuscript. MH and CD had primary responsibility for final content. All authors have read and approved the final manuscript.

## Funding

This study was supported by Pediatric endocrinology and diabetology unit, Lausanne University Hospital (Centre Hospitalier Universitaire Vaudois, CHUV). Funding was provided by Nestlé Research, Lausanne, Switzerland. Open access funding by University of Lausanne.

## Conflict of interest

CM, AE, AR, and CD are employed by Société des Produits Nestlé S.A.

The remaining authors declare that the research was conducted in the absence of any commercial or financial relationships that could be construed as a potential conflict of interest.

## Publisher’s note

All claims expressed in this article are solely those of the authors and do not necessarily represent those of their affiliated organizations, or those of the publisher, the editors and the reviewers. Any product that may be evaluated in this article, or claim that may be made by its manufacturer, is not guaranteed or endorsed by the publisher.

## Footnotes

^1^ https://valorinutritivi.ch/en/

^2^ https://fdc.nal.usda.gov/

^3^ https://www.iso.org/standard/43633.html
