# Supplementary figures and images for: Glucose variability in 6–12-month-old healthy infants
Source: Front Nutr. 2023 Jul 12;10:1128389. doi: 10.3389/fnut.2023.1128389 (PMC10369064; doi:10.3389/fnut.2023.1128389)

## Changes in glucose response to feeding in 6–12-month-old healthy infants

### Participant Flow Diagram

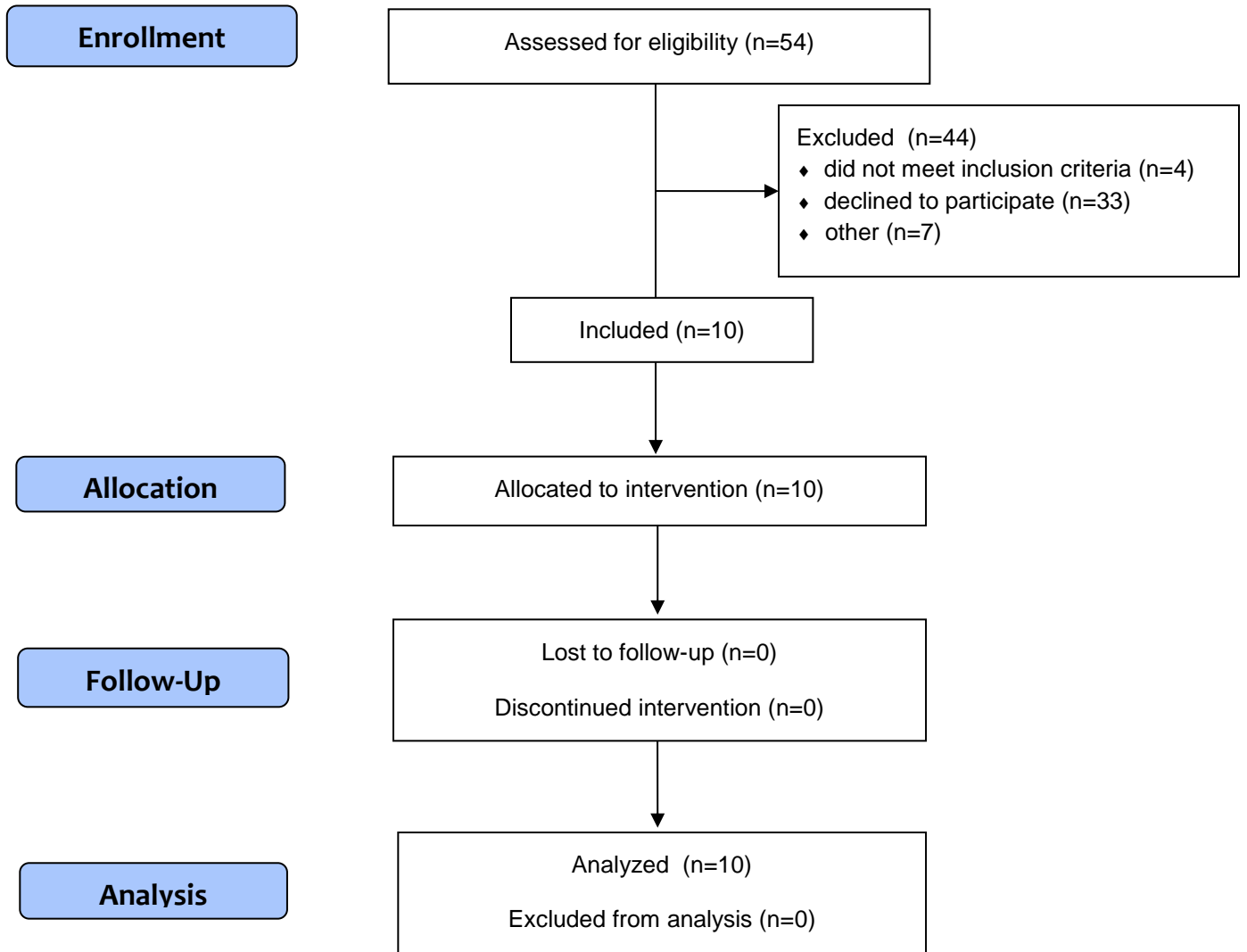

Supplement: Supplementary file 1 [file Data_Sheet_1.PDF]
